# An assessment of the quality of care for children in eighteen randomly selected district and sub-district hospitals in Bangladesh

**DOI:** 10.1186/1471-2431-12-197

**Published:** 2012-12-26

**Authors:** Dewan ME Hoque, Muntasirur Rahman, Sk Masum Billah, Michael Savic, AQM Rezaul Karim, Enayet K Chowdhury, Altaf Hossain, SAJ Md Musa, Harish Kumar, Sudhansh Malhotra, Ziaul Matin, Neena Raina, Martin W Weber, Shams El Arifeen

**Affiliations:** 1 International Centre for Diarrhoeal Diseases Research, Bangladesh (ICDDR,B), 68 Shaheed Tajuddin Ahmed Sarani; Mohakhali, Dhaka, 1212, Bangladesh; 2 Directorate General of Health Services, Government of Bangladesh, Dhaka, Bangladesh; 3 World Health Organization, Country Office, Dhaka, Bangladesh; 4 World Health Organization, Regional Office for South-East Asia, New Delhi, India; 5 World Health Organization, Country Office, Jakarta, Indonesia; 6 UNICEF, Dhaka, Bangladesh

**Keywords:** Quality of care, Hospitals, Child health, Bangladesh

## Abstract

**Background:**

Quality hospital care is important in ensuring that the needs of severely ill children are met to avert child mortality. However, the quality of hospital care for children in developing countries has often been found poor. As the first step of a country road map for improving hospital care for children, we assessed the baseline situation with respect to the quality of care provided to children under-five years age in district and sub-district level hospitals in Bangladesh.

**Methods:**

Using adapted World Health Organization (WHO) hospital assessment tools and standards, an assessment of 18 randomly selected district (n=6) and sub-district (n=12) hospitals was undertaken. Teams of trained assessors used direct case observation, record review, interviews, and Management Information System (MIS) data to assess the quality of clinical case management and monitoring; infrastructure, processes and hospital administration; essential hospital and laboratory supports, drugs and equipment.

**Results:**

Findings demonstrate that the overall quality of care provided in these hospitals was poor. No hospital had a functioning triage system to prioritise those children most in need of immediate care. Laboratory supports and essential equipment were deficient. Only one hospital had all of the essential drugs for paediatric care. Less than a third of hospitals had a back-up power supply, and just under half had functioning arrangements for safe-drinking water. Clinical case management was found to be sub-optimal for prevalent illnesses, as was the quality of neonatal care.

**Conclusion:**

Action is needed to improve the quality of paediatric care in hospital settings in Bangladesh, with a particular need to invest in improving newborn care.

## Background

Quality of care provided to children in hospital settings in low-income countries has generally been found to be poor [1-6]. A study of hospital quality for severely ill children across seven developing countries highlighted many of the quality of care issues [6].

The Government of Bangladesh (GoB) adopted the Integrated Management of Childhood Illness (IMCI) in 1998 as a key strategy to reduce child mortality and improve child development [7]. A recent review of IMCI implementation in Bangladesh and elsewhere highlighted the positive impact of IMCI on child health, quality of care, as well as inadequate referral of sick children from first-level and community care [8].

Studies in Ethiopia and Bangladesh have reported that 12-34% of children that come into contact with first-level facilities require referral to hospital care for further assessment and treatment [9,10]. The proportion needing hospital admission has been found to be even higher for infants [10]. The consequences of not accessing appropriate hospital care can be very serious and may include prolonged sickness and eventual death.

### Health system context in Bangladesh

In Bangladesh formal health services are provided by a range of public, private and non-government organization (NGO) providers. It has been estimated that there are approximately 4,400 of first level government facilities in Bangladesh [11].

The next tier of service is composed of some 417 sub-district hospitals called *Upazila Health Complexes*, which are designed to receive referrals from first level services. These usually have around 31–50 beds and are staffed by medical doctors, nurses, paramedics and laboratory technicians. There are also an estimated 59 district hospitals across the country each having between 50–250 beds [11].

### Quality improvement processes

The WHO framework for improving hospital care to children under five years of age outlines seven interconnected steps in the quality improvement process [12,13]. These include: 1) orientation to the specific country and health system context, and the adaptation of tools for assessment; 2) undertaking a baseline hospital assessment; 3) using this assessment to facilitate a consensus on hospital care and support system standards; 4) using standards to inform the development of appropriate interventions and improvement strategies; 5) implementing interventions and improvement actions; 6) monitoring and evaluating improvement actions; 7) sharing information about hospital improvement and successes to inform fine-tune existing strategies, develop new ones and scale-up hospital improvement [12].

The WHO has developed a number of generic assessment tools for evaluating the quality of hospital care to children; each of which has been developed based upon available evidence. The WHO *Assessment Tool for Hospital Care for Children* provides a set of standards for each of the most important dimensions of paediatric hospital care, and includes criteria for the assessment of performance in relation to each of these standards [12].

Given concerns about the quality of, and limited information about hospital care in Bangladesh, we undertook a baseline assessment of the quality of care for under five children in randomly selected government district and sub-district hospitals in Bangladesh as these are where severely ill children are most likely to be referred to. We also sought to compare the current practices in these hospitals with evidence-based guidelines, using the WHO developed pocket book of hospital care for children, to initiate quality improvement in the health sector [14]. In so doing we hoped to identify priority areas in need of improvement and to provide recommendations on strategies that could assist in this regard.

## Methods

### Selection of facilities

Six district hospitals from a number of districts across the country were randomly selected for inclusion in the assessment. All the districts in each of the six divisions in Bangladesh were assigned a number. A random number generator computer program was then used to randomly select one district from each division, and then a district hospital was chosen from each of the six randomly selected districts.

**Figure 1:**
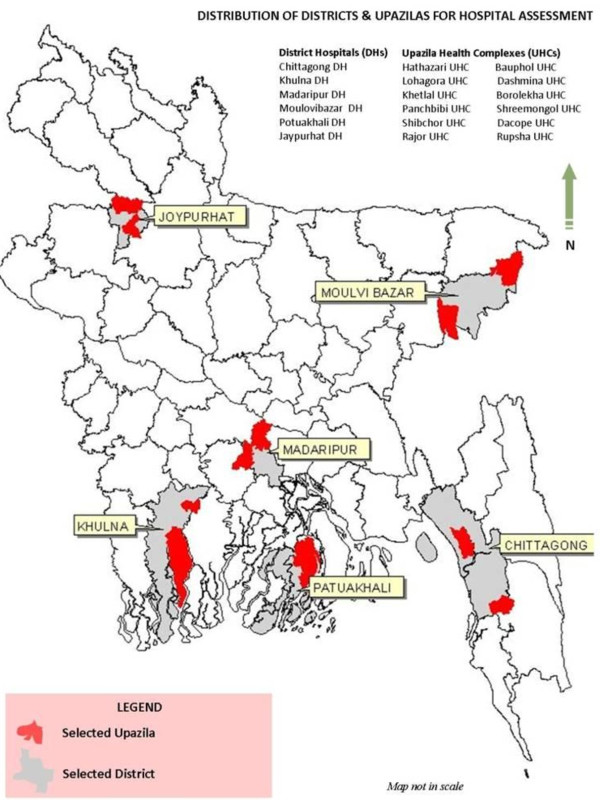
Distribution and districts and sub-districts (upazilas) for hospital assessment.

Twelve sub-district hospitals were also included in the assessment. Sub-district hospitals in the selected six districts were stratified in two strata- hospitals having IMCI program and hospitals having both IMCI and emergency obstetric (EmOC) program. One hospital from each of the strata was selected randomly, using the same procedure applied for district hospitals selection (Figure 1).

### Assessment tools

Adapted versions of the WHO developed generic assessment tools were used for our assessment. Adaptation of the tools was conducted through workshops involving leading paediatricians, hospital administrators and relevant government and NGO programme personnel in Bangladesh. The tool was then field tested prior to use in the study.

The final assessment tool included sections on hospital layout, essential drugs, supplies and equipment; laboratory support; emergency area and emergency management; neonatal care and case management for acute respiratory infections, diarrhoea, febrile illness and malnutrition; supportive care and monitoring; hospital administration; access to hospital care; and quality of care.

### Assessors

A total of 31 assessors were selected to undertake hospital assessments. Assessors were physicians and GoB and NGO programme personnel with experience in the area of child health. All assessors received three days training, facilitated by individuals who participated in the initial adaptation workshop. Assessors also participated in field practice sessions.

### Assessment of hospitals

Each team consisting of two paediatricians and two programme personnel conducted assessments at selected hospitals in May 2009. Assessments of each hospital took two days for teams to complete. Direct observation of clinical case management was undertaken wherever possible. When there were not sufficient patients for direct case observations, assessors reviewed clinical records. Assessors also conducted interviews with hospital staff and, if needed, used simulated cases when no case could be observed nor record could be found. The extraction of data from hospital Management Information System (MIS) reports was also used as another source of information.

Assessors scored hospitals on many of the dimensions in the adapted tool with respect to current WHO guidelines and standards, which were detailed in the tool. For individual and overall scoring, points from 5 to 1 were awarded: 5 for good practice complying with standards of care, 4 indicating little need for improvement to reach standard care, 3 meaning some need for improvement to reach standards of care, 2 indicating considerable need for improvement to reach standards of care and 1 for services not provided, or inadequate care and potentially life-threatening practices.

### Analysis

The results of completed hospital assessment tools were checked for consistency. A customized data entry programme was developed with the necessary consistency checks. After data entry and cleaning, the data was analyzed using SPSS version 12.

### Ethical consideration

Approval for health facility assessment and data collection was obtained from Directorate General of Health Services under the Ministry of Health and Family Welfare of Government of Bangladesh. Hospital assessment was an activity which documented the existing practice and situation at public health facilities. Since the assessment of the hospitals was conducted as part of quality of care improvement process at government hospitals, not as a research, we did not obtain ethical approval from ethics committee.

## Results

### Utilisation of hospitals by under-fives

**Figure 2:**
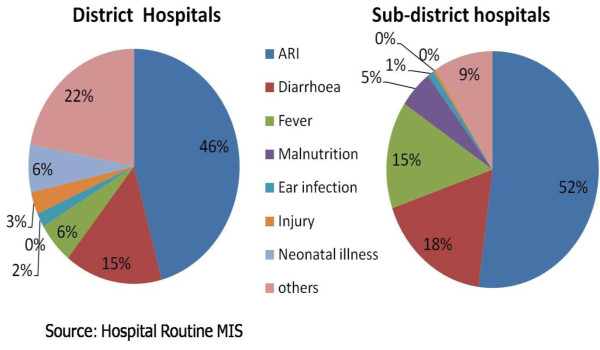
Major reasons for under-five visits to hospital in 2008.

Routine hospital MIS data showed that district hospitals (DHs) generally saw more children (M=17,435) than sub-district hospitals (SDHs) (M=8,728) in 2008. The proportion of children seen in emergency departments was greater in DHs (8.9%) as compared to SDHs (3.8%). A greater proportion of children were seen in outpatient departments in SDHs (86.3%) as compared to DHs (DHs: 80.8%), while the proportion of children seen in inpatient departments was similar (DHs: 10.3% and SDHs: 9.9%). The breakdown of hospital utilization by illness illustrated similar patterns for both district and sub-district hospitals (Figure 2). The most common illnesses in district hospitals (DHs) and sub-district hospitals (SDHs) were acute respiratory infections (DHs: 46% and SDHs: 52%), diarrhoea (DHs: 15% and SDHs: 18%), and fever (DHs: 6% and SDHs: 15%). Neonatal illness accounted for 6% of admissions in DHs but was negligible in SDHs.

### Hospital layout, staffing and child health infrastructure

All of the six DHs had a separate paediatric section and designated beds for children whereas seven SDHs had a separate paediatric section and only two had designated beds for children. None of the hospitals had paediatric surgeons. In terms of inpatient and outpatient care all DHs and SDHs had at least one doctor present during the morning shift (8 am-2 pm) but only a third of DHs (2 of 6) and two third of SDHs (8 of 12) hospitals had at least one doctor during the evening shift (2 pm-8 pm) and the night shift (8 pm-8 am). No DHs or SDHs had more than one doctor present in inpatient and outpatient departments during the evening and night shifts. All facilities had 24-hour nursing care for admitted children, and reported that emergency care provided by paramedics and/or on call doctors was available 24-hours a day and seven days a week. However, no hospitals had a functioning triage system for prioritizing severely ill children upon arrival at emergency departments (Table 1).

**Table 1:** Hospital layout and child health infrastructure

	**Number of district hospitals in which item was found (n=6)**	**Number of sub-district hospitals in which item was found(n=12)**
Separate paediatric section	6	7
Designated beds for children	6	2
Paediatric surgery facilities	3	3
Employment of paediatric surgeons	0	0
24-hour nursing care for children	6	12
Reported 24–7 availability of emergency services	6	12
Functioning triage system for emergency services	0	0

### Hospital support systems, equipment and essential drugs

Electricity was available at all hospitals, but amidst the back drop of frequent power cuts in Bangladesh, only one DH and four SDHs had a backup generator. Sufficient fuel to power backup generators was not available at any SDH. Whilst running water was available at all but one hospital, more than half (10 of 18 hospitals) were unable to provide safe drinking water. Five DHs and eight SDHs had coloured bins for waste disposal but instructions for correct waste disposal were not always followed (Table 2).

**Table 2:** Hospital support systems

	**Number of district hospitals in which item was found (n=6)**	**Number of sub-district hospitals in which item was found (n=12)**
Electricity available	6	12
Backup generator power	1	4
Sufficient fuel to power backup generators	1	0
Availability of running water	6	11
Provision of safe drinking water	4	6
Coloured bins for waste segregation and disposal	5	8

Functioning refrigerators for Expanded Programme on Immunization (EPI) vaccines were present at all hospitals. Filled oxygen cylinders were available but three SDHs lacked flow meters. Half of DHs (3 of 6) and a quarter of SDHs (3 of 12) had incubators for neonatal care, and only one hospital had a warmer. Weighing scales for children were available at the inpatient departments of almost all DHs (5 of 6) and in just over half of SDHs (7 of 12) (Table 3).

**Table 3:** Hospital equipment

	**Number of district hospitals in which item was found (n=6)**	**Number of sub-district hospitals in which item was found (n=12)**
Functioning refrigerators for vaccine storage	6	12
Filled Oxygen cylinders	6	12
Flow meters for Oxygen	6	9
Incubators for emergency neonatal care	3	3
Warmers for emergency neonatal care	1	0
Weighing scales in in-patient departments	5	7

Only one DH had all of the essential paediatric inpatient drugs (normal saline, cholera saline, injectable ampicillin, injectable gentamicin, syrup amoxicillin and injectable amoxicillin) whilst not a single SDH complied fully with the requirement. The majority of DHs (4 of 6) had a supply of normal saline, but this was present in less than half of SDHs (5 of 12). Cholera saline was found in almost all DHs and SDHs (5 and 11, respectively). Although both ampicillin and gentamicin should be available for the management of severe pneumonia or severe disease, at least one injectable antibiotic was available at all DHs, but not at all SDHs. Oral rehydration solution (ORS) and zinc tablets were available at all facilities (Table 4) .

**Table 4:** Availability of essential drugs

	**Number of district hospitals in which item was found (n=6)**	**Number of sub-district hospitals in which item was found (n=12)**
All essential paediatric drugs: normal saline, cholera saline, injectable (ampicillin, gentamicin and amoxicillin) and syrup amoxicillin	1	0
Injectable ampicillin	2	10
Injectable gentamicin	6	9
Injectable amoxixillin	2	5
Normal saline IV	4	5
Cholera saline	5	11
Tablet cotrimoxazole	6	12
Syrup amoxicillin	5	10
Oral rehydration solution	6	12
Zinc tablets	6	12

### Laboratory support

Supplies for essential laboratory tests, such as blood glucose, haemoglobin, complete blood count (CBC), blood grouping and cross-matching, routine microscopic examination for urine and stool and facilities for chest x-rays were present in most DHs and some SDHs. However, the presence of laboratory test was undermined by the availability of laboratory support, particularly in SDHs. For instance, laboratory test results were available on a 24/7 basis in just a quarter of SDHs (3 of 12). Similarly half of the DHs (3 of 6) and one third of the SDHs (4 of 12) were not conducting laboratory tests according to standard operating procedures, and maintenance of laboratory safety measures was generally poor (Table 5).

**Table 5:** Quality of laboratory support

	**Number of district hospitals in which item was found (n=6)**	**Number of sub-district hospitals in which item was found (n=12)**
24-7 availability and timely delivery of all essential laboratory tests (blood glucose, haemoglobin or haematocrit (PCV), microscopy for malaria, microscopy for cells in CSF and urine, blood grouping and cross-matching, HIV test)	5	3
Availability of supplies for blood glucose test	5	3
Availability of supplies for haemoglobin test	6	10
Availability of supplies for haematocrit (PCV) test	1	1
Availability of supplies for microscopy of malaria	4	3
Availability of supplies for microscopy for cells in cerebrospinal fluid	1	0
Availability of supplies for microscopy for cells in urine	6	11
Availability of supplies for blood grouping and cross-matching	6	3
Availability of supplies for HIV test	2	0
Performance of laboratory tests according to standard operating procedures	3	4
Maintenance of laboratory safety measures	2	3

### Availability and quality of neonatal care

Neither DHs nor SDHs had a separate unit or room for managing sick newborns. The safety of wards and hygiene practices relating to neonatal care was found to be poor overall. In particular substantial improvement was required in over half (7 of 12) of the SDHs in our sample. Likewise, no hospitals had adequate systems for prioritizing seriously ill neonates.

SDHs were found to be marginally better than DHs in promoting early initiation and exclusive breastfeeding and skin-to-skin contact for newborns (4 of 12 SDHs compared to 1 of 6 DHs). Adequate thermal protection for newborns was absent at all facilities and immunizations were not given routinely to all children born at the facilities.

Low birth weight (LBW) newborns were appropriately diagnosed at only two DHs. Treatment of LBW was poor at all hospitals, and particularly at SDHs, eight of which required substantial improvement compared to one DH. Similarly, appropriate feeding of young infants and LBW newborns was lacking at all hospitals (Table 6).

**Table 6:** Availability and quality of neonatal care

	**Number of hospitals where item was adequate**		**Number of hospitals where some improvement was needed**		**Number of hospitals where substantial improvement was needed**	
	**DH**	**SDH**	**DH**	**SDH**	**DH**	**SDH**
Hygienic and safe ward	0	2	5	3	1	7
Prioritization of seriously ill neonates	0	0	1	3	5	9
Promotion of early initiation of exclusive breastfeeding where skin contact is ensured	1	4	4	8	1	0
Practice of thermal protection	0	0	5	4	1	8
Administration of immunizations	0	1	6	6	0	5
Correct diagnosis of low birth weight newborns	2	1	3	6	1	5
Appropriate treatment of low birth weight newborns	0	0	5	4	1	8
Appropriate feeding of young infants and low birth weight newborns	0	0	3	3	3	9

### Quality of case management

Prevalent child illnesses such as pneumonia, diarrhoea and severe malnutrition were poorly managed at these facilities. This was despite the fact that all hospitals with the exception of one, had workers trained in IMCI. Even so, our assessment found that there was very little retraining, nor adequate and updated guidelines available at hospitals.

Severity of pneumonia was found to be correctly assessed and diagnosed in only one-third of DHs (2 of 6) and in one-quarter of SDHs (3 of 12). Appropriate antibiotics were found to be administered inadequately in all hospitals. Similarly correct and appropriate administration of oxygen was absent in most of the hospitals (Table 7).

**Table 7:** Quality of case management by illness

	**Number of hospitals where item was adequate**		**Number of hospitals where some improvement was needed**		**Number of hospitals where substantial improvement was needed**	
	**DH**	**SDH**	**DH**	**SDH**	**DH**	**SDH**
**Pneumonia**						
Correct assessment and diagnosis of the severity of pneumonia	2	3	2	4	2	5
Appropriate administration of antibiotics for pneumonia and other respiratory diagnoses	0	0	3	2	3	10
Correct administration of oxygen when necessary	1	0	3	8	2	4
Appropriate diagnosis and management of tuberculosis	4	1	2	8	0	3
**Diarrhoea**						
Correct assessment of dehydration	1	1	3	6	2	5
Administration and monitoring of rehydration plans appropriate to the severity of dehydration	0	0	4	4	2	8
Appropriate antibiotics administered only when necessary	0	0	5	2	1	10
Continued feeding given during diarrhoea	2	2	2	4	2	6
**Severe malnutrition**						
Nutritional status assessed by weight for height correctly, including differential diagnosis	0	0	1	1	5	11
Appropriate management of infection	0	0	3	6	3	6
Appropriate management of electrolyte imbalance and micronutrients	0	0	2	3	4	9
Correct management of dehydration	0	0	2	1	4	11
Hypoglycaemia and hypothermia correctly checked and managed	0	0	1	2	5	10
Correct feeding of severely malnourished children	0	0	1	2	5	10

Diagnosis and treatment of diarrhoea was also generally found to be deficient in most hospitals. Substantial improvement with respect to this was particularly required in SDHs (8 of 12) as compared to DHs (2 of 6). Despite two DHs and two SDHs meeting standards, continued feeding during diarrhoea was found to require improvement in most hospitals (Table 7).

All hospitals fell below standards in relation to severe malnutrition case management. Appropriate nutrition assessment for malnutrition was not conducted in many hospitals. As a result two thirds of DHs (4 of 6) and three quarters of SDHs (9 of 12) were found to need substantial improvement in managing electrolyte imbalance and micronutrients appropriately. Similarly correct management of dehydration, hypoglycaemia and hypothermia, and the correct feeding of severely malnourished children were lacking (Table 7).

### Patient monitoring system

Re-assessment of all admitted children by nurses was not conducted in any hospitals. A comprehensive monitoring chart has been developed by the Government of Bangladesh, which describes patient details, vital signs, clinical signs, treatments given, feeding practices, an intake-and-output chart, and outcomes of these practices. It however, was not used at most of the facilities (Table 8).

**Table 8:** Patient monitoring system

	**Number of hospitals where item was adequate**		**Number of hospitals where some improvement was needed**		**Number of hospitals where substantial improvement was needed**	
	**DH**	**SDH**	**DH**	**SDH**	**DH**	**SDH**
Re-assessment of all admitted children by a nurse	0	0	4	2	2	10
Re-assessment of all admitted children by a doctor	1	1	5	6	0	5
Assessment of nutritional status for all admitted children	0	0	2	1	4	11
Prioritization of severely ill children	1	0	3	5	2	7
Use of a monitoring chart for monitoring sick children	0	0	2	2	4	10

## Discussion

Consistent with research in other developing countries [1-6], we found that the quality of hospital care to under-fives in Bangladesh is poor and requires improvement. Deficiencies were found in a number of interconnected areas including in relation to triage systems, clinical case management and monitoring, the quality of neonatal care, essential hospital support systems and drugs, and hospital and child health infrastructure. Addressing these quality issues may not only improve the standard of care provided, but also improve access. For instance, Bari et al. found that perceptions of high quality care, alongside the availability of treatment, and the fact that care was free, were reasons cited by parents of newborns for accessing hospital care in one sub-district in Bangladesh [15].

Many deaths occur in the first 24 hours after arrival at a hospital and thus urgent intervention can be critical to child survival [16]. The WHO has developed guidelines for triage and emergency treatment of common emergency conditions that occur in under-five children in developing countries [6]. When accompanied by a short training course, the use of these guidelines by nurses was found to be effective in facilitating the identification and management of severely ill children in a hospital in Brazil [17].

This assessment found that out-patient case management and care practices were poor despite all but one hospital in our sample having IMCI-trained providers.

A study in Bangladesh highlighted the potential for incomplete assessments, and incorrect treatment amongst IMCI trained providers [18]. The reinforcement of training and provision of supportive supervision were factors that different studies identified as contributing to health workers performance in relation to IMCI [19,20].

Our findings illustrate that essential drugs, equipment and supports also need to be made available so that health providers can provide care based upon evidence-based guidelines. Even the best trained providers cannot treat severely ill children when, for instance, the essential drugs needed to do so are not at their disposal. Such essential supports were generally found to be lacking in the hospitals we assessed, which may be due to supply chain and stock management issues. While supply chain issues may take time to rectify, supervision may be a feasible way of improving stock management [21]. Similarly, inadequate laboratory support, as was found in many hospitals in this assessment, is likely to impact upon the quality of the diagnosis and treatment of severely ill children. Likewise a lack of equipment, as in the case of weighing machines for example, compromises proper assessment and leads to inappropriate treatment dosages given. English et al. document a similar situation in rural Kenya, in which they found between 30% to 56% of necessary items for paediatric care to severely ill children and newborns to be lacking in hospitals [1].

Although hospitals are supposed to function as places of healing and recovery, our findings point to the possibility that, if neglected, the hospital environment itself can have the opposite effect. Poor physical infrastructure such as unsafe drinking water or the lack of a back-up power supply found in many hospitals in our sample for instance, threaten to compromise patient safety.

The results of our assessment, when interpreted alongside the quality of care literature, [1-6] highlight the interconnectedness of the many dimensions that influence the quality of hospital care for children. In order to have the greatest impact on improving hospital quality, our findings suggests that it is not enough to focus solely on improving one dimension of hospital care alone whilst others remain neglected. For example increasing the training of hospital staff or providing them with refresher training is likely to increase clinical case management skills but is only likely to result in quality gains if the essential assessment and monitoring equipment, drugs, and laboratory support are present. And even if the necessary equipment and drugs are available to providers in order to optimize their clinical case management skills, positive outcomes for patients may be undermined by the hospital environment itself. Similarly even if improvements are made in other areas, deficient triage systems may mean that children most in need of urgent care do not receive the care they need. Therefore our assessment points to the need for an integrated package of quality improvement measures to be implemented. This would ideally include measures such as:

● Ongoing training, supportive supervision and guideline development to increase clinical case management skills of providers

● Investment in essential drugs, equipment, and laboratory supports to optimise the clinical case management skills of providers

● Investment in human resources, and in improving hospital infrastructure to ensure that the hospital environment is safe, and

● The development of triage and other systems based upon guidelines to prioritise those most in need.

● Regular feedback on hospital performance to motivate managers and workers, and sustain quality improvement

In Bangladesh particular emphasis and investment within an integrated quality improvement package needs to be placed on neonatal care. While some of the quality improvement measures that we have suggested may require significant investment and time to implement, others such as providing ongoing training, supportive supervision, guideline development and the development of triage systems in hospitals can be positive first steps. For instance, Ayieko et al. found that a multifaceted intervention, which included evidence-based clinical practice guidelines, training on emergency triage assessment and treatment, the provision of job-aides, local facilitation, and regular supervision and face to face feedback resulted in improvement in paediatric care in Kenyan district hospitals [22]. The authors also found that the implementation of the full intervention resulted in greater improvement in paediatric care practice as compared to a partial intervention, which included less quality improvement strategies. This reiterates the notion that multifaceted interventions may be more effective in improving the quality of care in resource poor settings than single interventions [23].

The persistence of quality of care issues in resource poor settings suggests that similar underlying issues may be present. Many of the issues that we and others have reported including poor hospital infrastructure, essential supports and drug availability may reflect health system deficiencies. But, as Travis et al. point out, health system strengthening has not been given adequate attention in comparison to specific disease focussed interventions [24]. Similarly, Rowe et al. argue that: *″There is a growing imperative to scale up delivery of key health interventions to meet the Millennium Development Goals. However, simply scaling up interventions in weak health systems that deliver poor quality services is likely to waste precious resources and fail to show the anticipated improvements in health″. (*pg 8) [23]. Because health system strengthening has been relatively neglected as part of the global health agenda, the potential for initiatives such as an integrated quality improvement approach to enhance system wide capacity is great.

National benchmarking or hospital accreditation may be possible ways in which an integrated quality improvement approach can occur in Bangladesh. Zambia has embarked upon a national hospital accreditation programme, in which hospitals undergo educational and accreditation surveys and are provided with feedback on how they can meet agreed-upon standards for accreditation [25]. This represents the next step in the hospital quality improvement process in Bangladesh.

An important foreseeable barrier to this pursuit is resource scarcity in developing countries like Bangladesh. However, one place to start might be a reassessment of the current allocation of resources. Carai et al. note that tertiary hospitals have tended to receive the large share of hospital funding even though the majority of severely ill children are treated in referral level facilities [26]. Whilst emphasis on primary care and prevention is needed, increasing investment in referral level facilities is also needed if child survival activities, such as IMCI, are to be successful.

## Conclusion

The results of this assessment are consistent with findings in the international literature and thus provide a strong rationale for quality improvement in Bangladeshi hospitals. Given the scale of neonatal mortality in Bangladesh, particular attention should be paid to improving neonatal care within such an integrated package.

## Competing interests

The authors declare that they have to competing interests.

## Authors’ contributions

DH contributed to the conception and design, adaptation of survey tools, field data collection, data processing and analysis, and the drafting and revisions of the paper. MR and MB contributed to the design, adaptation of data collection tools, data collection and analysis, drafting and revisions of the paper. RK and EK contributed to planning, field data collection, data analysis and revisions of the paper. MS contributed in data analysis and revision of the manuscript. AH and JM contributed in survey tools adaptation, trainings and supervised implementation of the data collection. HK, SM, ZM, NR, MW and SA contributed to the conception and design, prepared the draft of the paper and contributed to revisions of the paper. All authors critically revised the first draft for content and contributed to the final draft.

## Key messages

The quality of care for children in first level referral facilities in Bangladesh is poor.

Findings suggest that there is room for improvement in a number of interconnected areas including in relation to the layout and child health infrastructure in hospitals; hospital support systems, equipment and essential drugs; laboratory support; the availability and quality of neonatal care; and the quality of case management and patient monitoring systems.

An integrated quality improvement strategy is needed to improve the quality of care provided to children in district and sub-district hospitals in Bangladesh.

## Pre-publication history

The pre-publication history for this paper can be accessed here:

http://www.biomedcentral.com/1471-2431/12/197/prepub

## References

[B1] English M Ntoburi S Wagai J Mbindyo P Opiyo N Ayieko P Opondo C Migiro S Wamae A Irimu G An intervention to improve paediatric and newborn care in Kenyan district hospitals: understanding the context Implement Sci 2009 4 42 10.1186/1748-5908-4-42 19627588 PMC2724481

[B2] Reyburn H Mwakasungula E Chonya S Mtei F Bygbjerg I Poulsen A Olomi R Clinical assessment and treatment in paediatric wards in the north-east of the united republic of Tanzania Bull World Health Organ 2008 86 2 132 139 18297168 10.2471/BLT.07.041723 PMC2647389

[B3] English M Esamai F Wasunna A Were F Ogutu B Wamae A Snow RW Peshu N Delivery of paediatric care at the first-referral level in Kenya Lancet 2004 364 9445 1622 1629 10.1016/S0140-6736(04)17318-2 15519635

[B4] English M Esamai F Wasunna A Were F Ogutu B Wamae A Snow RW Peshu N Assessment of inpatient paediatric care in first referral level hospitals in 13 districts in Kenya Lancet 2004 363 9425 1948 1953 10.1016/S0140-6736(04)16408-8 15194254

[B5] Duke T Tamburlini G The Paediatric Quality Care Group Improving the quality of care in peripheral hospitals in developing countries Arch. Dis. Childh 2003 88 563 565 10.1136/adc.88.7.563 12818896 PMC1763179

[B6] Nolan T Angos P Cunha AJ Muhe L Qazi S Simoes EA Tamburlini G Weber M Pierce NF Quality of hospital care for seriously ill children in less-developed countries Lancet 2001 357 9250 106 110 10.1016/S0140-6736(00)03542-X 11197397

[B7] Tulloch J Integrated approach to child health in developing countries Lancet 1999 354 suppl. 2 16 20 10.1016/s0140-6736(99)90252-0 10507254

[B8] Ahmed HM Mitchell M Hedt B National implementation of integrated management of childhood illness (IMCI): policy constraints and strategies Health Policy 2010 96 2 128 133 10.1016/j.healthpol.2010.01.013 20176407

[B9] Simoes EAF Desta T Tessema T Gerbresellassie T Dagnew M Gove S Performance of health workers after training in integrated management of childhood illness in Gondar, Ethiopia Bull World Health Organ 1997 75 S43 53 PMC2487005 9529717

[B10] Kalter HD Schillinger JA Hossain M Burnham G Saha S de Wit V Khan NZ Schwartz B Black RE Identifying sick children requiring referral to hospital in Bangladesh Bull World Health Organ 1997 75 65 75 9529719 PMC2486991

[B11] Rahman RM Human rights, health and the state in Bangladesh BMC Int Health Hum Rights 2006 6 4 10.1186/1472-698X-6-4 16611360 PMC1513254

[B12] Campbell H Duke T Weber M English M Carai S Tamburlini G Global initiatives for improving hospital care for children: state of the art and future prospects Pediatrics 2008 121 4 e984 992 10.1542/peds.2007-1395 18381526 PMC2655645

[B13] WHO Improving the Quality of Hospital Care at First Referral Level 2001 Geneva: World Health Organization

[B14] WHO Pocket book of hospital care for children: Guidelines for the management of common illnesses with limited resources 2005 Geneva: World Health Organization 24006557

[B15] Bari S Mannan I Rahman MA Darmstadt GL Serajil MH Baqui AH El Arifeen S Rahman SM Saha SK Ahmed AS Trends in use of referral hospital services for care of sick newborns in a community-based intervention in tangail district, Bangladesh J Health Popul Nutr 2006 24 4 519 529 17591349 PMC3001156

[B16] Gove S Tamburlini G Molyneux E Whitesell P Campbell H Development and technical basis of simplified guidelines for emergency triage assessment and treatment in developing countries. WHO integrated management of childhood illness (IMCI) referral care project Arch Dis Child 1999 81 6 473 7 10.1136/adc.81.6.473 10569960 PMC1718149

[B17] Tamburlini G Di Mario S Maggi RS Vilarim JN Gove S Evaluation of guidelines for emergency triage assessment and treatment in developing countries Arch Dis Child 1999 81 478 482 10.1136/adc.81.6.478 10569961 PMC1718144

[B18] Arifeen SE Bryce J Gouws E Baqui AH Black RE Hoque DM Chowdhury EK Yunus M Begum N Akter T Siddique A Quality of care for under-fives in first-level health facilities in one district of Bangladesh Bull World Health Organ 2005 83 4 260 267 15868016 PMC2626213

[B19] Pariyo GW Gouws E Bryce J Burnham G Improving facility-based care for sick children in Uganda: training is not enough Health Policy Plan 2005 20 1 i58 i68 10.1093/heapol/czi051 16306071

[B20] Horwood C Vermaak K Rollins N Haskins L Nkosi P Qazi S An evaluation of the quality of IMCI assessments among IMCI trained health workers in south Africa PLoS One 2009 4 6 e5937 10.1371/journal.pone.0005937 19536288 PMC2693922

[B21] Trap B Todd CH Moore H Laing R The impact of supervision on stock management and adherence to treatment guidelines: a randomized controlled trial Health Policy Plan 2001 16 3 273 280 10.1093/heapol/16.3.273 11527868

[B22] Ayieko P Ntoburi S Wagai J Opondo C Opiyo N Migiro S Wamae A Mogoa W Were F Wasunna A Fegan G Irimu G English M A multifaceted intervention to implement guidelines and improve admission paediatric care in Kenyan district hospitals: a cluster randomized trial PLoS Med 2011 8 e1001018 10.1371/journal.pmed.1001018 21483712 PMC3071366

[B23] Rowe A de Savigny D Lanata C Victora C How can we achieve and maintain high-quality performance of health workers in low-resource settings? Lancet 2005 366 1026 1035 10.1016/S0140-6736(05)67028-6 16168785

[B24] Travis P Bennett S Haines A Pang T Bhutta Z Hyder AA Pielemeier NR Mills A Evans T Overcoming health-systems constraints to achieve the millennium development goals Lancet 2004 364 900 6 10.1016/S0140-6736(04)16987-0 15351199

[B25] Bukonda N Tavrow P Abdallah H Hoffner K Tembo J Implementing a national hospital accreditation program: the Zambian experience Int J Qual Health Care 2003 14 S7 16 10.1093/intqhc/14.suppl_1.7 12572783

[B26] Carai S Were W Weber M Hospital care for children in developing countries: getting the evidence where it matters Trop Med Int Health 2009 14 1324 1326 10.1111/j.1365-3156.2009.02390.x 19840348

